# Voltaic Narcotism

**Published:** 1860-03

**Authors:** 


*Voltaic Narcotism.*

Dr. Althaus, of London, in an article in the *Vienna Medical Weekly*, thus disposes of this subject as proposed by Dr. Richardson, and noticed in American Medical Monthly, Vol. XII., 312: It is well known that many experiments have been made to introduce medicinal agents into the organism by the help of galvanism, employing the *locomotive* force of the constant current by means of which liquids can be transported from one pole to another, without decomposition. Experiments on this subject were principally made by Sir Humphrey Davy, and more recently by Wiedemann. Experiments with the view of introducing medicinal agents may be considered as having failed, since the results claimed by Fabré-Palagret have not been confirmed by any one, and the experiments of Klencke and d'Hassenstein are generally doubted. Dr. Richardson claims, however, to introduce narcotic liquids in a portion of the body by the aid of electricity, and even to produce anæsthesia in this way. He styles this method *vol-*

*taic narcotism*, and, for some weeks, made considerable noise with it in the London hospitals, until Prof. Waller, of Birmingham, advanced the opinion in a lengthy article, that the anæsthesia produced in this way was simply due to the absorption of the narcotic substances. Dr. Richardson had stated that the local application of these substances, *without* electricity, never produced anæsthesia, not even when they were applied on a part as delicate as the ear of a rabbit. This is clearly not so, for in an experiment made by Dr. Althaus with chloroform and the constant current, complete anæsthesia of the skin was produced when pressure was employed for about ten minutes, a sponge being employed saturated with chloroform; the simultaneous action of the sponge and the poles of the galvanic battery did not, however, hasten the production of the anæsthetic effect. An hour after the experiment, he experienced a very acute pain, and on the next day there was developed an active inflammation, which lasted nine days, terminating in suppuration. During all this time the pain was severe, *(atroce,)* especially at night; the cicatrix formed slowly, first at the places where the chloroform alone had been applied, then at those where chloroform and galvanism had been used. Prof. Waller made experiments on animals with the narcotic solution, (equal parts of tincture of aconite and chloroform,) proposed by Richardson, and they died in a short time after the experiments, in consequence of poisoning of the blood by the narcotic solution. It is, then, possible that such a disastrous effect might be produced on children, especially such as were debilitated, if operations were practiced on them by the aid of voltaic narcotism. From all this, it follows that chloroform,* despite the attacks made upon it, is thus far the only means really useful, and relatively devoid of danger in the production of anæsthesia in chirurgical operations, and that all other means proposed to replace it have been demonstrated as insufficient.—*Wiener Med. Wochenschrift.*

